# Expression of IL-23 in gilt endometrium and oviduct after insemination with seminal plasma, spermatozoa or semen extender

**DOI:** 10.1186/s13104-021-05630-8

**Published:** 2021-06-03

**Authors:** Anna Svensson, Jatesada Jiwakanon, Caroline Fossum, Anne-Marie Dalin

**Affiliations:** 1 Division of Reproduction, Department of Clinical Sciences, Swedish University of Agricultural Sciences (SLU), Uppsala, Sweden; 2 Department of Biomedical Science and Veterinary Public Health, Swedish University of Agricultural Sciences (SLU), Uppsala, Sweden; 3 Present Address: Research Group for Animal Health Technology, Khon Kaen University, Khon Kaen, Thailand

**Keywords:** IL-23, mRNA expression, Gilt, Endometrium, Oviduct, Insemination

## Abstract

**Objective:**

Insemination with spermatozoa, seminal plasma and extender, cause a rapid inflammatory response in pig endometrium, characterized by an influx of neutrophils into the uterus. The transient inflammatory response to semen involves cytokine induction. Potential functions for Interleukin-23 (IL-23) in the inflammatory response to different insemination treatments were examined by studying mRNA expression and immunostaining in gilt oviduct and endometrium 35–40 h after insemination. Insemination was performed with seminal plasma (SP), spermatozoa (SPZ) without SP in the extender Beltsville thawing solution (BTS), or BTS alone. In control gilts an insemination catheter was inserted without anything being inseminated.

**Results:**

Results showed that IL-23 mRNA was expressed in oviduct and endometrium after insemination regardless of treatment. There was an approximate two- to fourfold increase in expression of IL-23 mRNA in catheter-insertion control compared with SPZ, SP and BTS treatment groups. IL-23 immunolabelling was detected in a small number of separate cells and in the sub-epithelial connective tissue of the endometrium, the endosalpinx of isthmus and infundibulum.

**Conclusion:**

In conclusion, insemination with SP, SPZ in BTS, and BTS alone decreased the expression of IL-23 mRNA in the endometrium compared to catheter-insertion control, indicating a possible role for IL-23 in the inflammatory response after insemination in gilts.

## Introduction

Insemination with spermatozoa, seminal plasma (SP) and extender inserted into the uterus via a catheter, causes a rapid inflammatory response in pig endometrium, characterized by neutrophil influx into the uterine lumen [1–3]. Increased neutrophil number may be due to chemotactic signals elicited by the insemination procedure [3, 4]. In pig oviduct, neutrophils were present in the connective tissue of the infundibulum about 40 h after insemination with semen in the extender Beltsville Thawing Solution (BTS) [5].

The transient inflammatory response to semen in pig reproductive tract involves induction of cytokines and chemokines [6]. Our previous immunohistochemical studies demonstrated presence of IL-6, IL-10 and TGF-β1 primarily in the epithelium of gilt oviduct [7] and endometrium [8] up to 40 h after insemination with SP, SPZ in BTS or BTS. TGF-β1 mRNA expression in gilt endometrium was lower 35–40 h after insemination with BTS or with SPZ in BTS compared with catheter-insertion controls [8]. Expression studies of pro-inflammatory and suppressive cytokines in porcine reproductive tract indicate complex patterns seemingly influenced by the insemination components. Therefore, archived samples from previous studies [7, 8] were analyzed for IL-23 expression. This cytokine promotes differentiation and proliferation of Th17 cells [9, 10]. Th17 cells stimulate various cells to produce cytokines and chemokines, with subsequent recruitment of neutrophils [9]. In human IL-23 regulates the function of decidual immune cells [11]. Weak IL-23 p19 immunolabelling has been detected in human endometrium [12].

The aim was to investigate a role for IL-23 in the inflammatory response to insemination by studying mRNA expression and immunostaining in gilt oviduct and endometrium 35–40 h after insemination with SP, SPZ in BTS, or BTS alone, compared to catheter-insertion controls.

## Main text

### Methods

#### Experimental design and insemination

Animal management, semen collection and insemination have been described earlier [7]. Semen from four fertile boars was collected and pooled. Motility of the spermatozoa was examined. Spermatozoa were isolated from seminal plasma using the single layer centrifugation technique [13].

Crossbred Landrace/Yorkshire gilts, 8–9 months old, from SLU´s experimental herd were inseminated once in their second or third oestrus at 15–20 h after the first signs of standing reflex (15–20 h before expected ovulation) with 100 mL of seminal plasma (SP, *n* = 4), spermatozoa in extender (100 mL BTS containing 5 × 10^9^ spermatozoa, SPZ, *n* = 4) [14] or BTS alone (*n* = 4). In four control gilts the insemination catheter (Goldenpig™, IMV, L’Aigle, France) was inserted without insemination of fluid (control, *n* = 4). The gilts were euthanized (stunned by captive bolt followed by exsanguination) 35–40 h after insemination. Uterine samples were collected from the mesometrial side, 20–30 cm from the tip of the uterine horn. Oviductal samples were collected from isthmus and from infundibulum. The study was approved by the Ethical Committee for Experimentation with Animals, Uppsala, Sweden (SLU.kv.Fe.2006.5.4.-15).

#### Quantitative real-time PCR

Frozen oviductal (isthmus and infundibulum) and endometrial samples, where the endometrium was separated from the myometrium, were homogenized. Total RNA was extracted with TRIzol® reagent (Invitrogen Ltd., Paisley, UK) and purified using the RNeasy Mini kit (Qiagen, Crawley, UK).. RNA content and purity was determined by 260/280 nm ratio using the NanoDrop® ND-1000 (Saveen & Werner AB, Limhamn, Sweden). All samples had ratios > 1.9. Nine random samples per experiment were checked for total RNA integrity with Agilent 2100 Bioanalyzer (Agilent Technologies, Waldbronn, Germany) [15]. cDNA was obtained using the iScript™ cDNA Synthesis Kit (Bio-Rad Laboratories, Hercules, CA) with 1 µg total RNA. Real-time PCR was performed using the Rotor-Gene 3000 (Corbett Life Science, Sydney, Australia) with iQ SYBR Green Supermix (Bio-Rad Laboratories, Hercules, CA). IL-23 primers (forward: 5′-TGTGGATCTACCAAGAGAAGAGG, reverse: 5′-AGGACTGACTGTTGTCCCTGA), were designed to amplify a cDNA fragment corresponding to nucleotides 168–277 of the pig IL-23 alpha subunit p19 mRNA sequence (GenBank Accession number NM_001130236). Hypoxanthine phosphoribosyl-transferase (HPRT) primers (forward: 5′-GTGATAGATCCATTCCTATGACTGTAGA, reverse: 5′-TGAGAGATCATCTCCACCAATTACTT [GenBank Accession number U69731]; [16]) and cyclophilin A primers [forward: 5′-TGCTTTCACAGAATAATTCCAGGATTTA, reverse: 5′-GACTTGCCACCAGTGCCATTA (GenBank Accession number AY266299)] were used for normalization. In each 25 µL reaction each primer concentration was 0.2 µM and 1 µL cDNA was added. Duplicate samples were submitted to amplification: 95 °C for 2 min, 40 cycles of 95 °C for 15 s and 60 °C for 1 min. Nonspecific amplification was eliminated by dissociation curves and no-template controls. Efficiencies (E) in a random sample (infundibulum; SP) were 111% (IL-23), 96% (HPRT) and 95% (cyclophilin A).

Threshold was adjusted to obtain the same C_t_ value for an inter-assay reference sample. IL-23 mRNA expression was normalized to HPRT and cyclophilin A mRNA expression according to: 2^Ct^ for IL-23/geometric mean of 2^Ct^ for HPRT and 2^Ct^ for cyclophilin A.

Results are presented as the inverted ratio (1/ratio) of the normalized expression [7].

#### Immunohistochemistry

Transverse (oviduct) or longitudinal (uterus) cryosections of 7 µm were blocked in 5% goat serum and incubated overnight at 4 °C with a rabbit anti-human IL-23 antibody (H-113; Santa Cruz Biotechnology Inc., Santa Cruz, CA) diluted to 0.2 µg/mL. Negative controls included sections incubated without primary antibody and sections incubated with ChromPure Rabbit IgG (011-000-003, Jackson ImmunoResearch Laboratories Inc., West Grove, PA). IL-23-like immunolabelling was detected using Vectastain® Elite ABC kit for Mouse IgG (PK-6102; Vector Laboratories Inc., Burlingame, CA) with biotinylated goat anti-rabbit IgG (BA-1000; Vector Laboratories Inc.) diluted 1/1000 replacing anti-mouse IgG. Endogenous peroxidase activity was blocked, 3,3′-diaminobenzidine tablets (D-5905; Sigma-Aldrich Inc., Saint Louis, MO) were used for visualization and sections were counterstained with Mayer’s Hematoxylin.

For semi-quantification of immunolabelling, photographs were taken with identical exposure settings. Immunolabelling intensity of endometrium and endosalpinx was scored (blind by three persons) from 0 to 4, where 0 corresponds to the intensity of the negative controls and 4 to very high intensity. The photographs were studied by eye and the intensity of the labelling was graded individually by each person.

#### Statistical analyses

Quantitative real-time PCR results are presented as mean values ± standard error of the mean (sem). Normalized mRNA expression data and IL-23-like immunolabelling intensity scores were analyzed using the SAS statistical package (version 9.1.3, SAS Institute, Inc., 2002–2003, Cary, NC). A natural log transformation was applied to the normalized copy number for analysis of variance. Differences in mean ratios were tested using analysis of variance (The Mixed Procedure). The statistical model included the fixed effect of treatment and tissue, the interaction between groups and tissue as well as the random effect of gilts nested within groups. The Bonferroni *t*-test was used to compare least-square mean values between groups when an overall significance for the effect was found. For statistical analysis of immunolabelling scores the NPAR1WAY procedure was used for standard analysis of variance, the Kruskal–Wallis test for one-way analysis of variance and the Wilcoxon rank sum test for non-parametric data for differences in mean scores. A value of p ≤ 0.05 was considered as statistically significant.

## Results

**Fig. 1:**
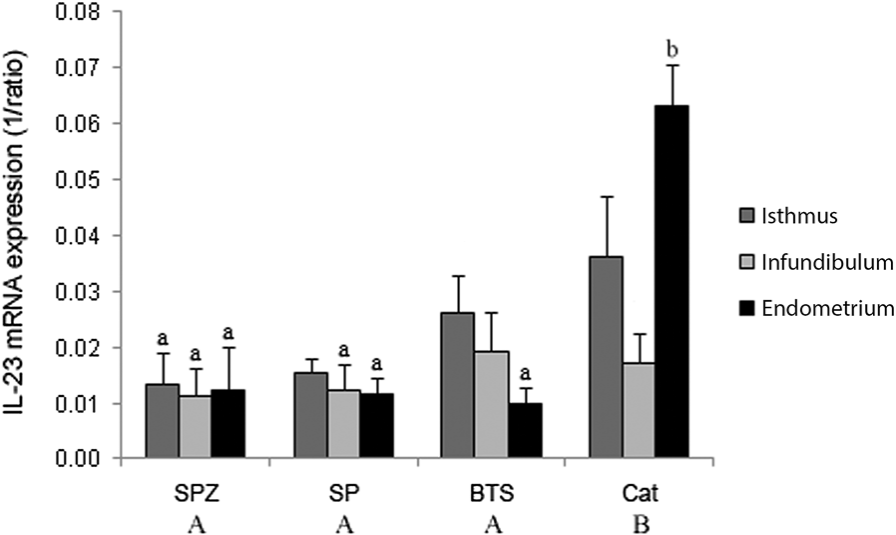
IL-23 mRNA expression in gilt oviduct and endometrium after insemination. IL-23 mRNA expression (mean 1/ratio ± sem) in gilt oviduct (isthmus and infundibulum) and endometrium at 35–40 h after insemination with spermatozoa in BTS (SPZ), seminal plasma (SP), BTS and catheter-insertion control (Cat; *n* = 4 per treatment group). Bars or treatment groups marked by different letters show significant difference (p ≤ 0.05). BTS; Beltsville thawing solution

Real-time PCR showed IL-23 mRNA to be expressed in gilt oviduct (isthmus and infundibulum) and endometrium 35–40 h after insemination regardless of treatment (Fig. 1). There were differences (p < 0.01) in IL-23 mRNA expression due to treatment. When all tissues within a treatment group were analyzed together, the SPZ in BTS, SP and BTS groups differed (p < 0.05) from the catheter-insertion control group but not from each other (NS). Interaction (p ≤ 0.05) between tissue and treatment is shown in Fig. 1. Endometrium from the catheter-insertion controls exhibited highest IL-23 mRNA expression. The effect of treatment was more pronounced in the endometrium than in the oviduct when tissues were analyzed separately. The results indicated a difference between individual animals.

**Fig. 2:**
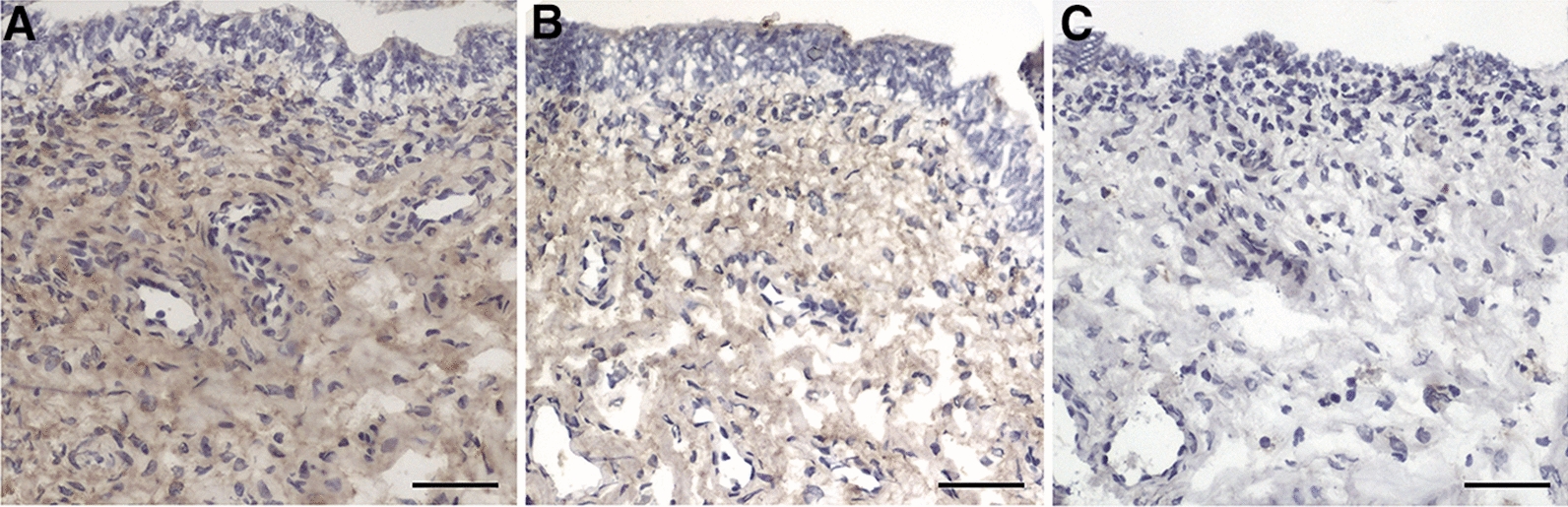
IL-23 immunolabelling in gilt endometrium at 35–40 h after insemination. Longitudinal sections of surface epithelium and sub-epithelial connective tissue of gilt endometrium (**A**–**C**). IL-23 immunolabelling in gilt endometrium after insemination with seminal plasma (**A**) or Beltsville thawing solution (BTS; **B**). **C** Negative control (rabbit IgG) shows no obvious staining of the endometrium 35–40 h after insemination with BTS. Representative pictures from each group are presented. Bars = 50 µm

**Fig. 3:**
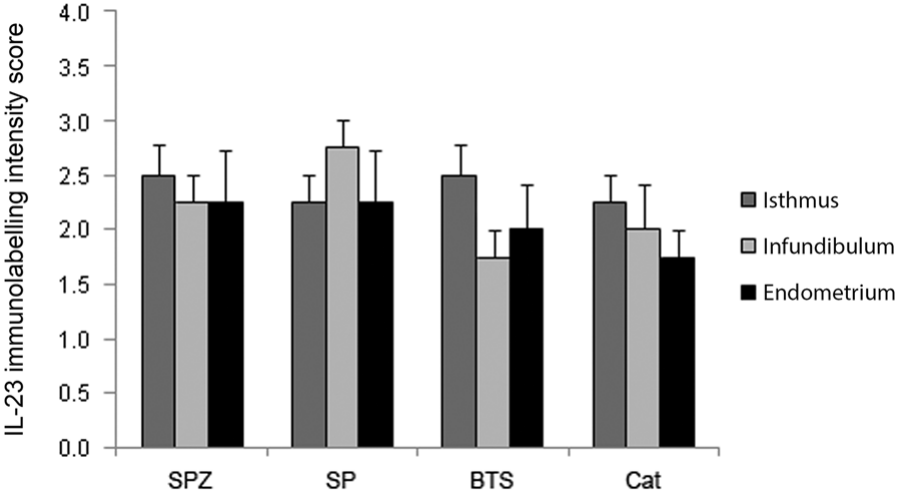
Intensity of IL-23 immunolabelling in gilt oviduct and uterus after insemination. IL-23 immunolabelling intensity scores (mean ± sem) in gilt oviduct (isthmus and infundibulum) and uterus at 35–40 h after insemination with spermatozoa in BTS (SPZ), seminal plasma (SP), BTS and catheter-insertion control (Cat; *n* = 4 per treatment group). *BTS* Beltsville thawing solution

IL-23 immunolabelling was found in the endometrium and in the endosalpinx of the isthmus and infundibulum. Staining was mainly found in the cytoplasm of the sub-epithelial connective tissue cells of the endometrium (Fig. 2A, B) and the same localization was observed in the oviduct (results not shown). Sections incubated with control IgG (endometrium; Fig. 2C) or with secondary antibodies only did not give any staining. Although IL-23 immunolabelling intensity scores differed between individual gilts, no significant differences were found between treatments or tissues (Fig. 3). A few distinct IL-23 immunolabelled cells were found in oviduct and endometrium in all treatment groups (results not shown).

## Discussion

IL-23 has earlier been studied in human endometrium but not in pig reproductive organs. The present study showed IL-23 mRNA to be expressed in oviduct and endometrium in gilts 35–40 h after insemination in all treatment groups. The sampling time point is estimated to 20–25 h after ovulation [2]. Our earlier studies [7, 8] revealed more clear treatment-dependent changes in cytokine mRNA expression at 35–40 h than shortly after insemination. In gilts/sows inseminated with semen, fertilized oocytes are then present in the oviduct [17]. The acute inflammatory reaction in the endometrium should decline to prepare for entering of early embryos [2]. Previous studies in gilts [7, 8] showed pro-inflammatory cytokine mRNAs (IL-6) to be differentially expressed at 35–40 h compared to shortly after insemination.

In this study, lower expression of IL-23 mRNA was seen in the SPZ, SP and BTS treatment groups, compared with the catheter-insertion control group, but no differences between the three insemination groups. The effect of treatment was more pronounced in the endometrium than in the oviduct. Endometrium from catheter-insertion controls exhibited the highest IL-23 mRNA expression. Components in the SPZ, SP and BTS treatments may exert some kind of suppression on IL-23 mRNA expression. To determine specific suppressive effects of treatment, inseminating animals with an equal volume of 0.9% NaCl would be interesting.

IL-23 promotes differentiation and proliferation of Th17 cells [9, 10] that are involved in inflammation by stimulating cytokine and chemokine production by various cells, with subsequent recruitment of neutrophils [10, 11, 18]. Increased levels of IL-23/IL-17 may be crucial for the diapedesis of neutrophils into tissues in pigs [18]. A role for IL-23 in recruiting neutrophils would be a possibility in gilt endometrium as seen after catheter insertion. In gilts of our previous studies [8, 17], neutrophils migrate into the endometrium in gilts inseminated with SPZ, whereas SP suppresses neutrophilic invasion and inflammation in the endometrium. The higher IL-23 mRNA expression in endometrium of catheter-insertion controls coincides in time (35–40 h) after treatment with higher presence of neutrophils compared with the SP group [8], indicating that SP may not only limit the neutrophil influx but also limit the expression of IL-23. More studies are needed to draw any conclusions about possible roles for IL-23 in porcine reproductive tissue.

Weak IL-23 p19 immunostaining has been shown in the cytoplasm of the sub-epithelial connective tissue cells in human endometrium [12]. Weak cytoplasm staining and low numbers of IL-23 immunolabelled cells were found in all treatment groups in endometrium and oviduct. These distinctly stained cells were too few for any conclusion to be drawn. The pattern for IL-23 is partly similar to TGF-β1 in endometrium showing higher expression for catheter than for BTS and SPZ but no difference compared with SP [8]. For the pro-inflammatory IL-6, the expression was low in all groups but lower for catheter than BTS [8]. SP from boar contain the suppressive cytokines IL-10 and TGF-β1 and low levels of IL-6 [19]. To our knowledge it is unknown if SP contains IL-23.

The present study shows IL-23 mRNA to be expressed in oviduct and endometrium in gilts 35–40 h after insemination. IL-23 immunolabelling was present in subepithelial connective tissue of endometrium and oviduct and in low numbers of immune cells. The results demonstrate a possible role for IL-23 in the immune response of reproductive tissue.

## Limitations

Animals inseminated with an equal volume of 0.9% NaCl may be desirable as a control in future studies.

## Data Availability

The datasets used and/or analysed during the current study are available from the corresponding author on reasonable request.

## Abbreviations

BTS: Beltsville thawing solution
Cat: Catheter
E: Efficiency
HPRT: Hypoxanthine phosphoribosyl-transferase
IL-17: Interleukin 17
IL-6: Interleukin-6
IL-10: Interleukin-10
IL-23: Interleukin-23
sem: Standard error of the mean
SP: Seminal plasma
SPZ: Spermatozoa
TGF-β1: Transforming growth factor-β1
Th17: T helper 17
